# Chemical characterization of non‐psychoactive *Cannabis sativa* L. extracts, in vitro antiproliferative activity and induction of apoptosis in chronic myelogenous leukaemia cancer cells

**DOI:** 10.1002/ptr.7357

**Published:** 2022-02-02

**Authors:** Lisa Anceschi, Alessandro Codeluppi, Virginia Brighenti, Riccardo Tassinari, Valentina Taglioli, Lucia Marchetti, Luca Roncati, Andrea Alessandrini, Lorenzo Corsi, Federica Pellati

**Affiliations:** ^1^ Department of Life Sciences University of Modena and Reggio Emilia Modena Italy; ^2^ Clinical and Experimental Medicine PhD Program University of Modena and Reggio Emilia Modena Italy; ^3^ National Laboratory of Molecular Biology and Stem Cell Bioengineering‐Eldor Lab National Institute of Biostructures and Biosystems (NIBB), Innovation Accelerator, CNR Bologna Italy; ^4^ Department of Experimental, Diagnostic and Specialty Medicine (DIMES) Alma Mater Studiorum University of Bologna Bologna Italy; ^5^ Institute of Pathology University of Modena and Reggio Emilia Modena Italy; ^6^ Department of Physics, Informatics and Mathematics University of Modena and Reggio Emilia Modena Italy; ^7^ National Research Council (CNR) CNR‐Nanoscience Institute‐S3 Modena Italy; ^8^ Biostructures and Biosystems National Institute (INBB) Inter‐University Consortium Rome Italy

**Keywords:** anticancer activity, cannabidiol, cannabinoids, *Cannabis sativa* L., leukaemia

## Abstract

In this study, extracts from non‐psychoactive *Cannabis sativa* L. varieties were characterized by means of ultra high‐performance liquid chromatography coupled with high‐resolution mass spectrometry (UHPLC‐HRMS) and their antiproliferative activity was assessed in vitro. The human chronic myelogenous leukaemia cell line K562 was chosen to investigate the mechanism of cell death. The effect on the cell cycle and cell death was analysed by flow cytometry. Proteins related to apoptosis were studied by western blotting. Mechanical properties of cells were assessed using the Micropipette Aspiration Technique (MAT). The results indicated that the cannabidiol (CBD)‐rich extract inhibited cell proliferation of K562 cell line in a dose‐dependent manner and induced apoptosis via caspase 3 and 7 activation. A significant decrease in the mitochondrial membrane potential was detected, together with the release of cytochrome c into the cytosol. The main apoptotic markers were not involved in the mechanism of cell death. The extract was also able to modify the mechanical properties of cells. Thus, this hemp extract and its pure component CBD deserve further investigation for a possible application against myeloproliferative diseases, also in association with other anticancer drugs.

## 1 INTRODUCTION

Cancer is a multifactorial disease encompassing a combination of factors, such as genetic susceptibility, exposure to carcinogens, random gene mutations and chronic inflammation. Whereas the first cause of death in the world is still due to cardiovascular diseases, recent studies have reported that cancer is the leading cause of death in high‐income countries (HIC), such as some western countries (Dagenais et al., 2020). If the trend reported in the study is confirmed, in a couple of decades cancer could be the first cause of death throughout the world. These data are of great importance and they deserve a very close consideration in both the medicinal chemistry and pharmacology ambits. Indeed, it seems paradoxical that in the HIC, where the anticancer treatments are both available and outstanding, the situation is so dramatic. Actually, due to the intrinsic cancer molecular features, the cure of the disease is still far from being achieved, although the great progress in the research and the latest anticancer‐drug discoveries. So, it becomes fundamental to investigate every possible new path leading to an improvement in current care.

In recent years an increasing number of studies have described the broad spectrum of biological activity of *Cannabis* extracts and cannabinoids (Muscarà et al., 2021a; Muscarà et al., 2021b), in particular as possible anticancer agents (Zhelyazkova, Kirilov, & Momekov, 2020), acting through several molecular mechanisms, such as induction of apoptosis, inhibition of angiogenesis and metastatic processes (Śledziński, Zeyland, Słomski, & Nowak, 2018). Kis et al. (2019) have described the ability of cannabidiol (CBD) to modulate the tumorigenesis in a different type of cancers. Cannabinoids increase the apoptotic response in leukaemia, supporting the overall concept that these compounds, and in particular CBD, can be valid therapeutic agents. The activity of CBD appears to be mediated by the interaction with G protein‐coupled receptor 55 (GPR55) and the transient receptor potential cation channel subfamily V member 1 (TRPV1) (Muller, Morales, & Reggio, 2019), but not with the classical cannabinoid receptors CB_1_ and CB_2_ (Pellati, Borgonetti, et al.,  2018). CBD can also directly target mitochondria and perturbs calcium homeostasis in acute lymphoblastic leukaemia (Olivas‐Aguirre et al., 2019).

CBD is the main compound present in non‐psychoactive or fibre‐type *Cannabis sativa* L., commonly known as hemp, which differs from recreational and medicinal chemotypes for its cannabinoid profile (Aliferis & Bernard‐Perron, 2020). Indeed, hemp is a rich source of non‐psychoactive cannabinoids, contrary to recreational and medicinal *Cannabis* varieties, which are characterized by a remarkable high content of the psychoactive compound ∆^9^‐tetrahydrocannabinol (∆^9^‐THC), together with its acidic precursor ∆^9^‐tetrahydrocannabinolic acid (∆^9^‐THCA) as well as cannabinolic acid (CBNA) and cannabinol (CBN), the last two being the oxidized products of ∆^9^‐THCA and ∆^9^‐THC, respectively (Aliferis & Bernard‐Perron, 2020; Appendino, Chianese, & Taglialatela‐Scafati, 2011). In general, the main cannabinoids present in hemp inflorescences are cannabidiolic acid (CBDA) and its decarboxylated form CBD, but other cannabinoids can be also found, though in much lower content, including cannabigerolic acid (CBGA), cannabigerol (CBG), cannabichromenic acid (CBCA) and cannabichromene (CBC) (Appendino et al., 2011). Even if medical *Cannabis* and its psychoactive peculiar compound Δ^9^‐THC remain the main object of research work, scientific studies devoted to unravelling the biological potential of hemp and non‐psychoactive cannabinoids have recently increased in number (Corsi, Pellati, Brighenti, Plessi, & Benvenuti, 2019; Iseppi et al., 2019; Muscarà et al., 2021a; Muscarà et al., 2021b; Pellati, Borgonetti, et al., 2018; Pellati, Brighenti, et al., 2018).

As the exploration of natural therapeutics continues to expand, non‐psychoactive cannabinoids represent a powerful source for complementary therapeutics of cancer diseases (Roncati, Vadalà, Corazzari, & Palmieri, 2021). As a result of their antiproliferative properties, in the appropriate context they could be also applied as standalone cancer treatments. In this ambit, this work aimed was to evaluate the antiproliferative activity of standardized extracts obtained from hemp inflorescences belonging to different varieties against human cancer cells of different embryological origin. In this context, the importance of the use of a phytocomplex in therapy should be considered. Indeed, the presence of different compounds in plant extracts often allows for a better management and control of possible side effects. The activity of the hemp extracts was investigated on the possible modulation of the cell cycle regulation, the involvement of apoptosis and the regulation of biomechanical properties of cell membranes as well.

## 2 MATERIALS AND METHODS

### 2.1 Chemicals and solvents

Stock methanolic solutions (1.0 mg/ml) of, cannabidiol (CBD), cannabigerol (CBG) and cannabinol (CBN) and stock acetonitrile solutions (1.0 mg/ml) of cannabidiolic acid (CBDA) and cannabigerolic acid (CBGA) were purchased from Cerilliant Corporation (Round Rock, TX, USA). CBD, CBG and CBN stock solutions were stored at −20°C, while CBDA and CBGA solutions were stored at −80°C.

Acetonitrile (ACN), absolute ethanol (EtOH), methanol (MeOH), formic acid (HCOOH), dimethyl sulfoxide (DMSO), 10,000 UI penicillin ‐ 10 mg/ml streptomycin association, cell counting kit‐8 and imatinib were purchased from Sigma Aldrich s.r.l. (Milan, Italy). Water (H_2_O) was purified by using a Milli‐Q Plus185 system from Millipore (Milford, MA, USA). Dulbecco's Modified Eagle's Medium (DMEM) was purchased from PAN Biotech (Aidenbach, Germany). Roswell Park Memorial Institute (RPMI) media were from EuroClone s.p.a. (Milan, Italy). Bio Whittaker foetal bovine serum (FBS) was acquired from Lonza. Glutamine was from Invitrogen s.r.l. (San Giuliano Milanese, MI, Italy).

### 2.2 Hemp plant material

Hemp plant material was kindly provided by the research centre CREA‐CIN (Rovigo, Italy) from plants varieties cultivated in open field under the same growing conditions. In particular, female inflorescences from three different hemp varieties listed within the EU database of admitted agricultural species (Carmagnola lot n° CAR35‐2017, Santhica lot n° SAN22‐2017 and Bernabeo lot n° BER17‐20107) were chosen as the starting material for the preparation of the extracts, given their high content of non‐psychoactive cannabinoids (Brighenti, Pellati, Steinbach, Maran, & Benvenuti, 2017; Pellati, Brighenti, et al., 2018).

### 2.3 Preparation of raw hemp extracts

For each hemp variety, 50 g of grounded hemp inflorescences were added with an initial aliquot of 300 ml EtOH and then submitted to dynamic maceration at room temperature for 15 min. The extract was then paper filtered and the residue was extracted with the same procedure three times with an additional 200 ml of the extraction solvent. The extracts were combined and then brought to dryness under vacuum with a Heidolph Laborota 4,000 WB rotary evaporator (Schwabach, Germany). Finally, the extract was placed in a desiccator under vacuum until it reached a constant weight. Extracts obtained from raw hemp inflorescences are referred in the text as R1 (Carmagnola), R2 (Santhica) and R3 (Bernabeo). For R1, R2 and R3 the percentage yield was 7.3, 5.6 and 6.7%, respectively. Approximately 25 mg of the extract were dissolved in 25 ml of the extraction solvent and the solution was injected into the HPLC system after filtration through a 0.45 μm PTFE filter.

#### 2.3.1 Preparation of decarboxylated hemp extracts

A weighed amount of grounded hemp inflorescences (15 g) was placed in an oven at a temperature of 110°C for 15 min. The temperature was then raised to 120°C and kept constant for 60 min. The decarboxylated inflorescences were then submitted to dynamic maceration with EtOH, following the same procedure as that described in the previous section. Extracts obtained from decarboxylated hemp inflorescences are indicated in the text as D1 (Carmagnola), D2 (Santhica) and D3 (Bernabeo). For D1, D2 and D3 the percentage yield was 3.0, 2.8 and 3.1% respectively. In this case also, a known amount of the extract (~ 25 mg) was dissolved in 25 mL of EtOH and then filtered through a 0.45 μm PTFE filter prior to the injection into the HPLC system.

### 2.4 UHPLC‐HRMS analysis of hemp extracts

Qualitative analysis of hemp extracts was performed by means of ultra high‐performance liquid chromatography coupled with high‐resolution mass spectrometry (UHPLC‐HRMS). The analyses were performed on a Thermo Scientific (Massachusetts, United States) UHPLC Ultimate 3,000 equipped with a vacuum degasser, a binary pump, a thermostatted autosampler, a thermostatted column compartment and a Q‐Exactive Orbitrap mass spectrometer with a heated electro‐spray ionization (HESI) source. An Ascentis Express C_18_ column (150 mm × 3.0 mm I.D., 2.7 μm, Supelco, Bellefonte, PA, USA) was used. A ternary (A/B/C) multistep gradient (solvent A: 0.1% HCOOH in H_2_O, solvent B: 0.1% HCOOH in ACN and solvent C: MeOH) was used. On the basis of a previous work (Berman et al., 2018), the multistep gradient program was established as follows: 0–2 min from 50 to 67% B which was kept for 4 min, 6–10 min from 67 to 90% B which was kept for 4 min, 14–15 min from 90% to 50% B, which was kept for 5 min for re‐equilibration of the system prior to the next injection. Solvent C was constant at 5% throughout the run. A flow rate of 0.3 ml/min was used. The column temperature was 30°C. The injection volume was 1 μl.

MS acquisition was carried out with a heated electro‐spray ionization source operated in both the positive and the negative ion mode. As to the MS detector, the source parameters were set as follows: sheath gas (N_2_) 40, auxiliary gas (N_2_) 30, auxiliary gas temperature 290°C, electrospray voltage 3.5 kV (+) and 3.2 kV (−). The analyses were acquired in the full mass data‐dependent (FM‐dd‐MS/MS) mode at a resolving power of 35.000 full width at half maximum (FWHM). The other mass analyzer parameters were set as follows: scan range *m*/*z* 150–2000, automatic grain control (AGC) target 1 × 10^6^ ions in the Orbitrap analyzer, ion injection time 100 ms and isolation window for the filtration of the precursor ions *m*/*z* 1.0. The fragmentation of precursors ions was performed at 20, 30 and 50 as normalized collision energies (NCE).

### 2.5 HPLC‐UV analysis of hemp extracts

The HPLC‐UV analyses of the extracts were performed on an Agilent Technologies (Waldbronn, Germany) modular model 1260 Infinity II system, consisting of a quaternary pump, a manual injector and a UV variable wavelength detector. Chromatograms were recorded by using an Agilent OpenLab ChemStation (Rev. C.01.10). An Ascentis Express C_18_ column (150 mm × 3.0 mm I.D., 2.7 μm, Supelco, Bellefonte, PA, USA) was used, with a mobile phase composed of 0.1% HCOOH in both (A) H_2_O and (B) ACN. The gradient elution was as follows: 0–13 min 60% B, 13–17 min from 60 to 80% B, 17–22 min from 80 to 90% B, which was kept for 8 min. The post‐running time was 15 min. The flow rate was 0.4 ml/min. The sample injection volume was 3 μl. Chromatograms were acquired at 210 nm (for decarboxylated cannabinoids) and at 220 nm (for cannabinoic acids). Calibration curves for target compounds were constructed at five calibration levels by plotting the peak areas of the analytes vs. their concentration.

The value corresponding to the limit of quantification (LOQ) was 2.5 μg/ml for each cannabinoid, while the limit of detection (LOD) was 0.8 μg/ml.

Two injections were performed for each sample.

### 2.6 GC‐FID analysis of hemp extracts

GC‐FID analysis of volatile compounds in the hemp extracts were carried out on a 7820A GC coupled with a flame ionization detector (FID) from Agilent Technologies. Compounds were separated on an Agilent Technologies HP‐5 cross‐linked poly‐5% diphenyl–95% dimethyl polysiloxane (30 m × 0.32 mm i.d., 0.25 mm film thickness) capillary column. The column temperature was initially set at 45°C, then increased at a rate of 2°C/min up to 100°C, then raised to 250°C at a rate of 5°C/min, and again raised up to 280°C at a rate of 11°C/min, and finally held for 15 min. The injection volume was 0.1 μl in the split mode 1:20 (v/v). Helium was used as the carrier gas at a flow rate of 1.0 ml/min. The extracts were diluted to 1:20 (v/v) with *n*‐hexane before GC‐FID analysis. The analyses were performed in triplicate for each sample.

### 2.7 Preparation of the solutions used in the biological assays

Stock solutions of fibre‐type hemp extracts and CBD were prepared by dissolving a known amount of the extract/compound (3 and 100 mg, respectively) in 0.1–1.0 ml of DMSO. The working solutions were obtained by the dilution of the stock solution in the culture medium to reach the desired concentration. The content of DMSO in the working solutions was below 0.5%.

### 2.8 Cell cultures

Human colorectal adenocarcinoma HT29 cell line was grown in DMEM medium with high glucose without sodium pyruvate supplemented with 10% FBS, 2 mM L‐glutamine, 100 U/ml penicillin and 100 μg/ml streptomycin. Human glioblastoma U87MG cell line was grown in DMEM medium with high glucose and with sodium pyruvate supplemented with 10% FBS, 2 mM L‐glutamine, 100 U/ml penicillin and 100 μg/ml streptomycin. Human chronic myelogenous leukaemia K562 cell line was grown in RPMI medium supplemented with 10% FBS, 2 mM L‐glutamine, 100 U/ml penicillin and 100 μg/ml streptomycin.

All the cell lines were obtained from the American Type Culture Collection (ATCC, Teddington, UK). The cells were cultured in a humidified incubator at 37°C with 95% humidity and 5% CO_2_. All cell lines were used between the 10^th^ and 30^th^ passage.

### 2.9 Cell viability measurement

Cell viability was assessed after 24, 48 and 72 hr of continuous exposure with the different compounds described above. Cell viability of K562, U87MG and HT29 were evaluated by means of the Cell Counting Kit‐8 (CCK‐8) (Dojindo Laboratories, Kumamoto, Japan). Briefly, the cells were plated on 96‐well plates (Euroclone, Milan, Italy) at concentration of 5,000 or 10,000 cells/cm^2^. After exposure to desired concentrations of the different compounds, 10 μl of CCK‐solution was added to each well and incubated for a period of 2 hr at 37°C. The absorption was measured at 450 nm using a multiplate reader multiscan FC (Thermo Scientific, USA). Cell viability was expressed as a percentage of that of the untreated cells referred as control. Cell viability assays were performed in triplicate with *n* = 4 per group.

### 2.10 UHPLC‐HRMS analysis of CBD and its oxidation products in cell lysates

For the identification of CBD, monomeric and dimeric CBD hydroxyquinone in lysates, K562 cells treated for 24 hr with D1 extract at 20 μg/ml were pooled, resuspended in MeOH and mechanical disrupted by repeated freezing cycles. For the UHPLC‐HRMS analysis of lysates the mobile phase, gradient elution and flow rate were the same used for the HPLC‐UV analysis (Section 2.5). A targeted‐SIM data‐dependent MS^2^ analysis was performed in both the positive and the negative ion mode. As to the MS detector, the source parameters were set as follows: sheath gas (N_2_) 45, auxiliary gas (N_2_) 25, auxiliary gas temperature 290°C and electrospray voltage 3.8 kV. The analyses were acquired at a resolving power of 35.000 full width at half maximum (FWHM). The other mass analyzer parameters were set as follows: scan range *m*/*z* 200–800, automatic grain control (AGC) target 2 × 10^5^ ions in the Orbitrap analyzer, ion injection time 60 ms and isolation window for the filtration of the precursor ions *m*/*z* 1.0. The fragmentation of precursors ions was performed at 20, 40 and 70 as normalized collision energies (NCE).

The control of the online analyses was carried out using Xcalibur 3.0 software (Thermo Fisher Scientific, San Jose, CA, USA).

### 2.11 Analysis of cell cycle and BrdU/PI

The evaluation of the effect on the different phases of the cell cycle was assessed by BrdU/PI staining performed as described by Manfredini et al. (1997). K562 cells were seeded at a density of approximately 6 × 10^5^ cells/well into 24‐well plates, cultured 16 hr with D1 extract 20 μg/ml. Following 16 hr of incubation, cells were pre‐incubated with 10 μM BrdU (Sigma Aldrich, St Louise, MO, USA) and stained with a purified mouse primary monoclonal antibody (MoAb) directed against BrdU (BD Biosciences, Erembodegem, Belgium), followed by a rabbit anti‐mouse immunoglobulin IgG secondary antibody conjugated with fluorescein isothiocyanate (FITC) (Dako A/S, Glostrup, Denmark). Samples were then re‐suspended in a 50 μg/ml PI water solution. The assays were analysed by Attune NxT flow cytometer (Thermofisher Scientific, Massachusetts, USA).

### 2.12 Annexin V/PI assay

Annexin V/PI staining assay was performed according to the supplier instructions (BD, Milan, Italy). Briefly, K562 cells (50.000 cells/ cm^2^) treated with the D1 extract at the indicated concentrations for 16 hr were harvested, washed in PBS and re‐suspended in Annexin V binding solution at the final concentration of 1 × 10^6^ cells/ml. A total of 100 μl of this suspension was transferred into a new tube, to which 5 μl Annexin V (FITC conjugated) and 5 μl propidium iodide (PI) were added. The reaction was carried out for 15 min at room temperature. The stained cells were analysed directly by Attune NxT flow cytometer (Thermofisher Scientific, Massachusetts, USA).

### 2.13 Immunoblotting

Total and nuclear proteins were extracted from K562 cells using NE‐PER nuclear and cytoplasmic extraction reagents (Thermo Fisher Scientific, Massachusetts, USA), according to the manufacturer protocol. As regards the analysis of cytochrome c presence, subcellular fractionation was performed as described by Dimauro, Pearson, Caporossi, and Jackson (2012) in order to separate using a SCEB gradient the cytosolic and mitochondrial fractions. Lysate proteins were quantified using the Bradford colorimetric method Comassie (Thermo Fisher Scientific, Massachusetts, USA), according to the manufacturer protocol. Equal amounts of proteins, 20 μg for each sample, was loaded into a pre‐cast 4–12% Bis‐Tris Plus (Invitrogen, Milan, Italy) and electrophoretically transferred to a nitrocellulose membrane (Invitrogen, Milan, Italy). Membrane was blocked in TBST (20 mMTris‐ HCl, 0.5 M NaCl and 0.05% Tween 20) buffer containing 5% non‐fat dried milk at room temperature for 3 hr under gentle agitation and incubated with primary antibody anti‐p53 (1:1000), anti‐bcl‐2 (1:1000), anti bcl‐xl (1:1000) anti‐GAPDH (1:1000), anti‐cytochrome c (1:1000) overnight at 4°C (the primary antibodies were from Cell Signalling, USA). Membrane was then washed three times in TBST, incubated for 1 hr with HRP‐conjugated anti‐rabbit or anti‐mouse antibody (Cell Signalling, USA) and visualized using a chemiluminescence method (Amersham, GE Healthcare Europe GmbH, Milan, Italy).

The immune‐complexes were analysed using densitometric analysis. The determination of relative protein expression was performed using a BioRad GS 690 Imaging densitometer with molecular analysis software (Life science, Milan, Italy) with GAPDH as the loading control.

### 2.14 Caspase assay

To evaluate caspase ‐ induced cell death, the CellEvent Caspase‐3/7 Green Detection Reagent® (Invitrogen #C10423) was used. Briefly, cells were seeded at a concentration of 5,000 cells per square centimetre in 48 wells, using a complete RPMI medium with 10% FBS, 1% of glutamine and 1% of pen/strep antibiotics. Next day, cells were treated with 30 μg/ml of D1 and immediately added with the ready to use fluorogenic substrate to a final concentration of 2 μM, without a washing step. After an incubation of 30 min at 37°C and 5% CO_2_, protected from light, cells were imaged under the microscope for 48 hours inside an OKOlab on‐stage incubator in a controlled environment of 37°C, constant humidity and 5% of CO_2_, protected from light.

Cells were visualized with a Nikon Inverted Microscope Eclipse Ti‐E, equipped with a Digital Sight camera DS‐Qi2 (Nikon Instruments, Tokyo, Japan) and images were acquired with NIS‐Elements (Nikon Instruments, Tokyo, Japan) software. The whole well was imaged at each time point with automatic stitching performed by the software. Four experiments were performed for each data set and caspase ‐ activated positive cells were counted on six fields of the same size.

### 2.15 Mitochondrial membrane potential evaluation

To evaluate mitochondrial membrane potential, JC1 was used (Invitrogen #T3168). Briefly, cells were first stained for 30 min at 37°C and 5% CO_2_, protected from light, using JC1 at a concentration of 5 μM in RPMI medium. Cells were then seeded at a concentration of 5,000 cells per square centimetre in 48 wells, using complete RPMI medium and treated with 30 μg/ml of D1 extract.

Cells were immediately imaged under the microscope for 24 hr inside an OKOlab on‐stage incubator in a controlled environment of 37°C, constant humidity and 5% of CO_2_, protected from light. Cells were visualized with Nikon Inverted Microscope Eclipse Ti‐E equipped with a Digital Sight camera DS‐Qi2 (Nikon Instruments, Tokyo, Japan) and images were acquired with NIS‐Elements (Nikon Instruments, Tokyo, Japan) software. Six experiments were performed for each data set and JC1 ratiometric evaluation was done comparing the evolution of fluorescence signal in the two channels of emission (polarized vs. depolarized mitochondria) over time.

### 2.16 Assessment of the effect on cell mechanics using the micropipette aspiration technique (MAT)

Capillaries with an external diameter of 1 or 1.5 mm were bought from World Precision Instruments (WPI, Sarasota, FL, USA). BSA for glass surface passivation (both pipettes and cell chambers) was purchased from Sigma‐Aldrich (Sigma‐Aldrich, St. Louis, MO, USA). Micro‐aspiration was performed, as previously described (Di Cerbo et al., 2018). During all the process, images of cells were acquired at a rate of 1 frame per sec by an Olympus IX 70 inverted microscope in Differential Interference Contrast (DIC) mode with a 20x objective. The ImageJ software (NIH, Washington, USA) was then used to automatically detect the position of the cell protrusion.

### 2.17 Statistical analysis

Graphs showed in the figures represent the mean of the three independent experiments, with an error bar indicating the standard deviation. Differences between the treated cells versus control cells were analysed using the GraphPad Prism 8.0 software (GraphPad Software, Inc., San Diego, CA, USA). Statistical comparison was performed by applying one‐way analysis of variance (ANOVA) and Dunnett's multiple‐comparison test. *p* values <.05 were considered significant.

## 3 RESULTS AND DISCUSSION

### 3.1 Chemical composition of hemp extracts

HPLC has been widely applied for the qualitative and quantitative analysis of cannabinoids in *C. sativa* extracts (Brighenti et al., 2017; Brighenti et al., 2021; Pellati, Brighenti, et al., 2018; Protti et al., 2019). As for sample preparation from the plant material, polar solvents, such as EtOH, under dynamic maceration at room temperature have been found to provide the highest extraction efficiency for both cannabinoic acids and decarboxylated cannabinoids (Brighenti et al., 2017) and, therefore, this procedure was applied in this work.

In this study, the identification of the secondary metabolites in hemp ethanolic extracts was carried out by UHPLC‐HRMS. In order to verify the presence of additional cannabinoids in hemp for which analytical standards were not available, a target metabolomic analysis was used. This was performed using a compiled list of 213 cannabinoids (Table S1), on the basis of those described in the literature (Berman et al., 2018; Thomas & ElSohly, 2016) and additional potential cannabinoids that were added according to the biosynthetic pathways described in the literature (Appendino et al., 2011; Berman et al., 2018; Thomas & ElSohly, 2016). Decarboxylated extracts were fully characterized by UHPLC‐HRMS, as they showed to be more active on the tested cancer cell lines. The MS and MS/MS data for the decarboxylated extracts are shown in Table 1. Cannabinoic acids showed a higher intensity of the signals in the negative ion mode, as opposed to the neutral compounds which ionized better in the positive ion mode.

**TABLE 1:** Chemical formula, retention time, MS and MS/MS data of the compounds identified in decarboxylated hemp extracts (D1‐D3) both in the positive and negative ion mode by UHPLC‐HRMS

Compound	Formula	*t*_*R*_ (min)	Precursor ion (m/z)	Product ion (m/z)	D1	D2	D3
Positive ion mode							
Cannflavin B	C_21_H_20_O_6_	5.8	369.1333	315.2315	✓	✓	✓
Cannabinodiol (CBND)	C_21_H_26_O_2_	8.7	311.2006	293.1898	✓		
Cannabidivarin (CBDV)	C_19_H_26_O_2_	9.0	287.0060	277.2160	✓		
Hydroxycannabidiol (OH‐CBD)	C_21_H_30_O_3_	10.3	331.2268	313.2160	✓	✓	✓
Cannflavin A	C_26_H_28_O_6_	10.8	437.1959	359.2211	✓	✓	✓
Cannabigerol (CBG)	C_21_H_32_O_2_	11.4	317.2475	245.1547	✓	✓	✓
Cannabidiol (CBD)	C_21_H_30_O_2_	11.6	315.2319	245.1547	✓	✓	✓
Epoxycannabigerol	C_21_H_32_O_3_	8.4/11.7	333.2424	315.2319		✓	✓
Cannabinol (CBN)	C_21_H_26_O_2_	13.1	311.2006	293.1898	✓		
Δ^9^‐Tetrahydrocannabinol (∆^9^‐THC)	C_21_H_30_O_2_	13.6	315.2321	245.1547	✓		
10‐Oxo‐Δ^6a^‐Tetrahydrocannabinol‐C5 (OTHC)	C_21_H_28_O_3_	13.9	329.2112	315.2318	✓	✓	
Cannabichromene (CBC)	C_21_H_30_O_2_	14.9	315.2321	245.1547	✓		✓
Negative ion mode							
Cannabielsoin acid (CBEA)/Hydroxycannabidiolic acid (OH‐CBDA)	C_22_H_30_O_5_	4.9/9.4	373.2028	345.2076	✓	✓	✓
Cannabidiolic acid (CBDA)	C_22_H_30_O_4_	10.8	357.2021	313.2176	✓	✓	✓
Cannabigerolic acid (CBGA)	C_22_H_32_O_4_	11.2	359.2228	331.2285	✓	✓	✓
Δ^9^‐Tetrahydrocannabinolic acid (Δ^9^‐THCA)	C_22_H_30_O_4_	13.6	357.2021	313.2177	✓		
Cannabichromenic acid (CBCA)	C_22_H_30_O_4_	16.1	357.2021	313.2177	✓		

In the positive ion mode, ∆^9^‐THC was detected in the D1 extract only. The same was for CBN and CBND, which may be present in the extract as metabolites of ∆^9^‐THC. In the negative ion mode, both ∆^9^‐THCA and CBCA were detected in the D1 extract only (Table 1).

**FIGURE 1:**
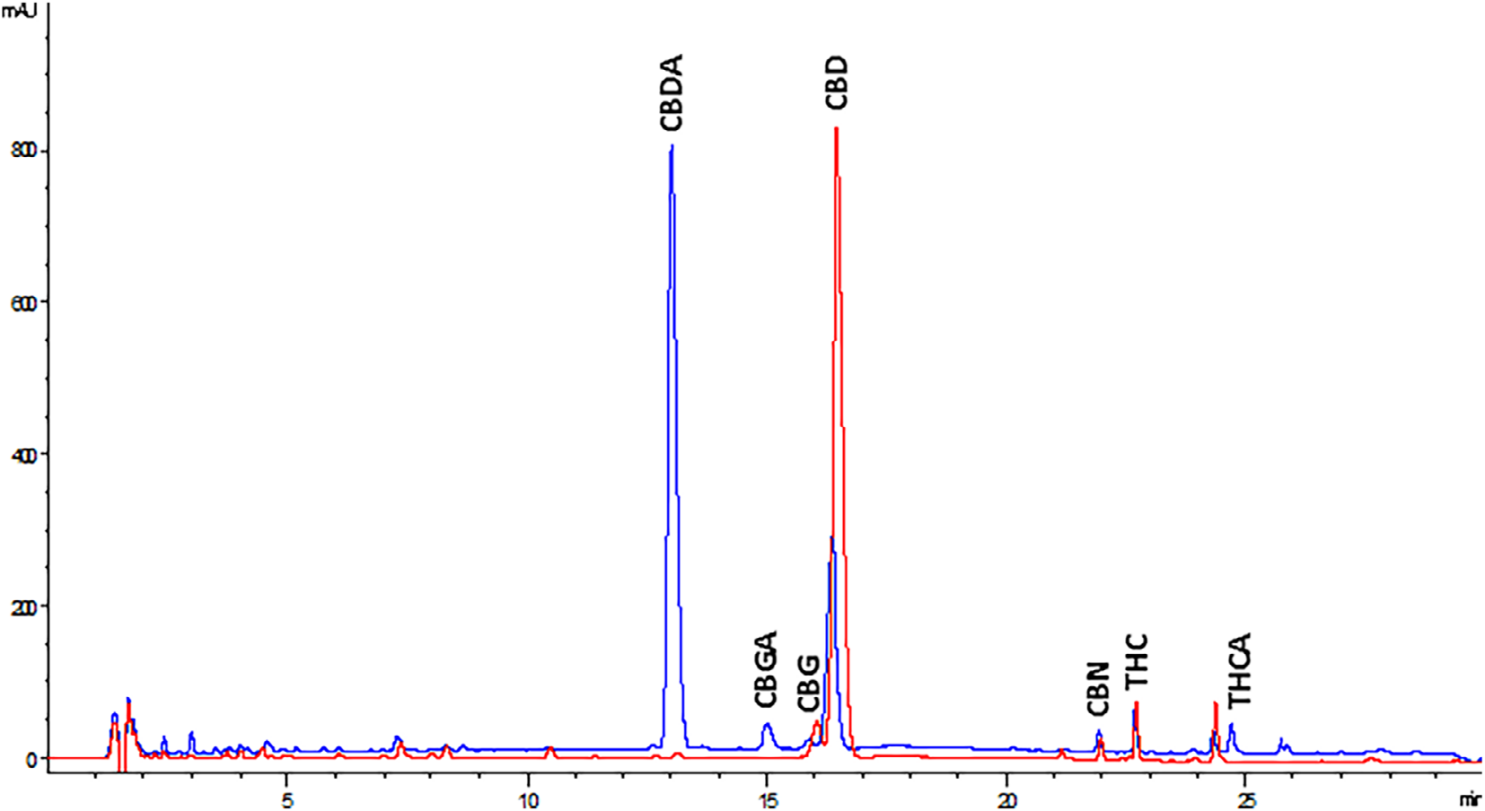
Representative HPLC‐UV overlapped chromatograms of R1 (blue) and D1 (red) extracts at 220 nm. The main neutral and acid cannabinoids are shown

Figure 1 shows representative HPLC‐UV overlapped chromatograms of R1 (blue) and D1 (red) extracts at 220 nm. The raw extract R1was rich in CBDA; its chromatogram also shows minor peaks belonging to CBGA and ∆^9^‐THCA. As regards the decarboxylated extract D1, the HPLC‐UV analysis revealed that the decarboxylation process had successfully occurred. HPLC‐UV chromatograms of the other extracts are reported in the Data S1 (Figure S1).

Table 2 shows the quantitative results obtained from the HPLC‐UV analysis of cannabinoids both in the raw (R1‐R3) and in decarboxylated (D1‐D3) hemp extracts. The quantification of the main cannabinoids in the extracts was achieved by an external calibration with pure analytical standards and the calibration curves were linear for all the analytes (*r* ^2^ > 0.998). The amounts of cannabinoids determined in the extracts from the decarboxylated plant material of the Carmagnola variety (D1) agrees with what has been previously described in the literature (Corsi et al., 2019). In the D1 extract, CBD reached up to 92% of total cannabinoids, while the percentage of CBG and ∆^9^‐THC was 4 and 1% respectively. In sample D2, CBD and CBG correspond to 70 and 27% of total cannabinoids, respectively. In D3, CBD and CBG represented 30 and 68% of total cannabinoids, respectively.

**TABLE 2:** Results of the HPLC‐UV analysis of cannabinoids in raw (R1‐R3) and decarboxylated hemp extracts (D1‐D3)

Peak number	Compound	*t*_*R*_ (min)	R1	R2	R3	D1	D2	D3
1	CBDA	11.6	306.4 ± 0.4	63.7 ± 1.5	8.3 ± 0.8	<LOQ	<LOQ	<LOQ
2	CBGA	13.4	15.4 ± 1.4	105.9 ± 1.2	68.1 ± 0.2	<LOQ	<LOQ	<LOQ
3	CBG	14.9	3.9 ± 0.1	74.1 ± 0.6	75.8 ± 0.6	19.7 ± 2.5	48.0 ± 0.5	160.9 ± 1.8
4	CBD	15.4	134.1 ± 2.3	58.7 ± 1.8	57.8 ± 0.8	440.8 ± 5.0	125.8 ± 2.4	70.2 ± 1.1
5	CBN	19.8	8.2 ± 0.4	<LOQ	3.2 ± 0.1	6.9 ± 0.3	2.9 ± 0.2	3.5 ± 0.6
6	∆^9^‐THC	21.6	<LOQ	<LOQ	<LOQ	5.3 ± 0.1	<LOQ	<LOQ
7	∆^9^‐THCA	23.6	11.5 ± 1.5	<LOQ	<LOQ	<LOQ	<LOQ	<LOQ

*Note*: LOQ, limit of quantification. Data are expressed as mg/g ± SD.

As regards polyphenols, cannflavins A and B were detected in the positive ion mode, as shown in Table 1. These compounds are known to be present in very low amount in hemp extracts, as previously described in detail (Pellati, Brighenti, et al., 2018).

Moreover, the presence of residual terpenes in the extracts was evaluated by means of GC‐FID analysis. The results showed that both raw and decarboxylated extracts did not contain residual volatile compounds, such as mono‐ and sesquiterpenes (Iseppi et al., 2019), due to the high temperature used during the decarboxylation process.

### 3.2 Antiproliferative activity of a CBD‐rich hemp extract on different human cancer cell lines

**FIGURE 2:**
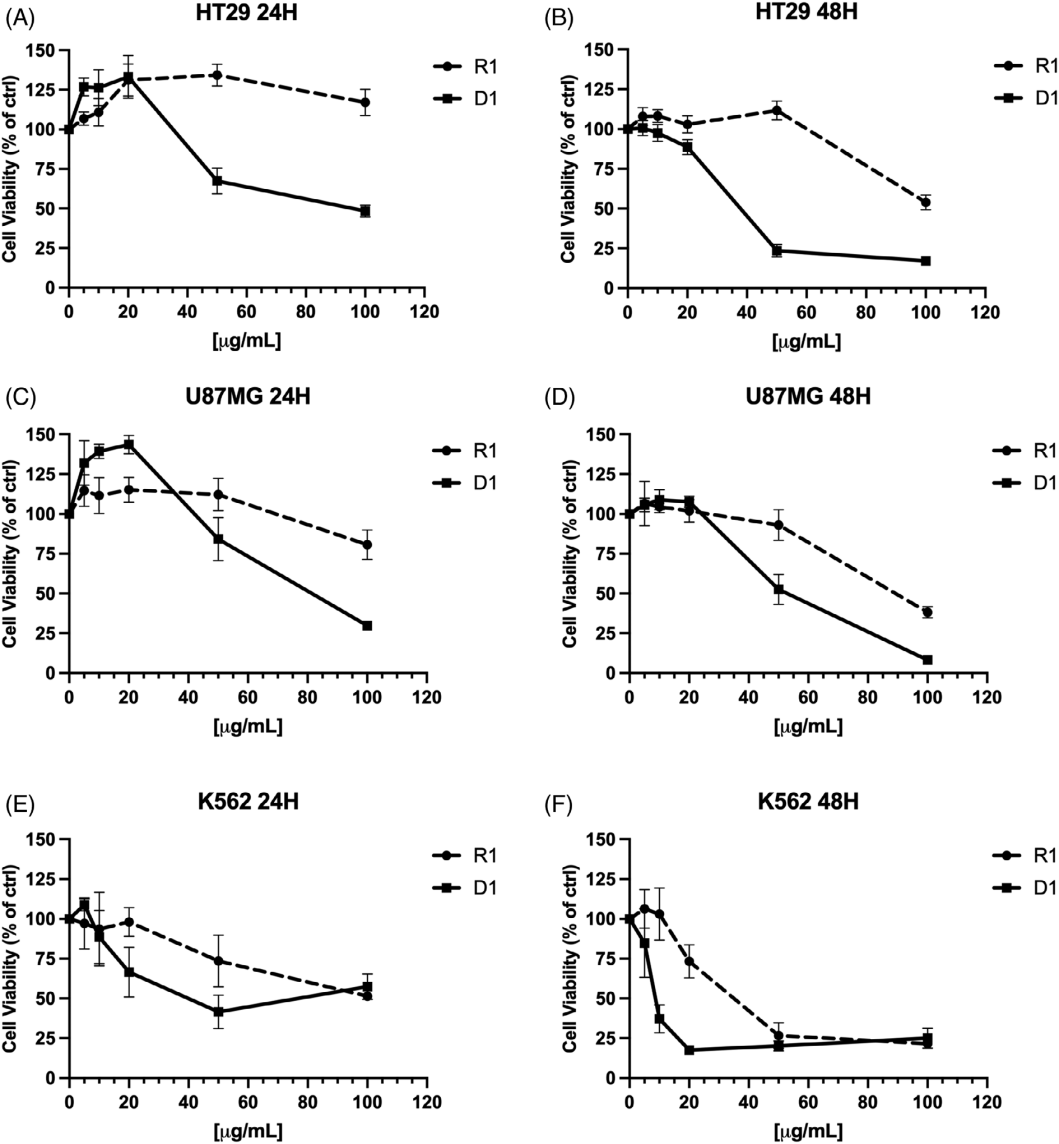
Viability of HT29 (A,B), U87MG (C,D) and K562 (E,F) cells treated at 24–48 hr with raw (R1) and decarboxylated (D1) CBD‐type hemp extracts at 5–100 μg/ml. Data in the graphs are shown as mean ± SD (*n* = 4 per group)

The potential ability of the CBD‐rich extracts R1 and D1 to interfere with cell viability was assessed at 24 and 48 hr on different human cancer cell lines, including the coloncarcinoma HT29, the glioblastoma U87MG, and the myeloid chronic leukaemia K562. For the first screening, we decided to test the CBD‐type hemp variety. This choice was based on the recent findings by Petrovici et al. (2021) on the antiproliferative activity of CBD on cancer cells. After 24 hr of treatment, the R1 extract from raw CBD‐type hemp inflorescences did not alter cell viability in all the cell lines tested, except at the highest concentration of 100 μg/ml (Figure 2a,c,e). Instead, the D1 extract obtained from the decarboxylated plant material showed to be much more active in all the tested cell lines, reaching a significant inhibition of cell viability on K562 and U87MG at a concentration of 70–80 μg/ml (Figure 2c,e).

This effect was more pronounced after 48 hr of treatment for both the extracts in all the cell lines (Figure 2b,d,f). Interestingly, K562 cells were the most sensitive to the treatment with R1 and D1 (Figure 2f). In particular, D1 was able to reduce the K562 cell viability by about 50% and 80% at 10 μg/ml and 20 μg/ml concentrations, respectively (Figure 2f). Imatinib, used as a positive control in comparison with the extracts, showed an IC_50_ of 1 μM on K562 cells after 48 hr of treatment. These initial findings led us to choose the chronic myelogenous leukaemia K562 as a suitable model to further characterize the antiproliferative activity of hemp extracts with a different phytochemical composition.

### 3.3 Antiproliferative activity of different hemp extracts on K562 cells

**FIGURE 3:**
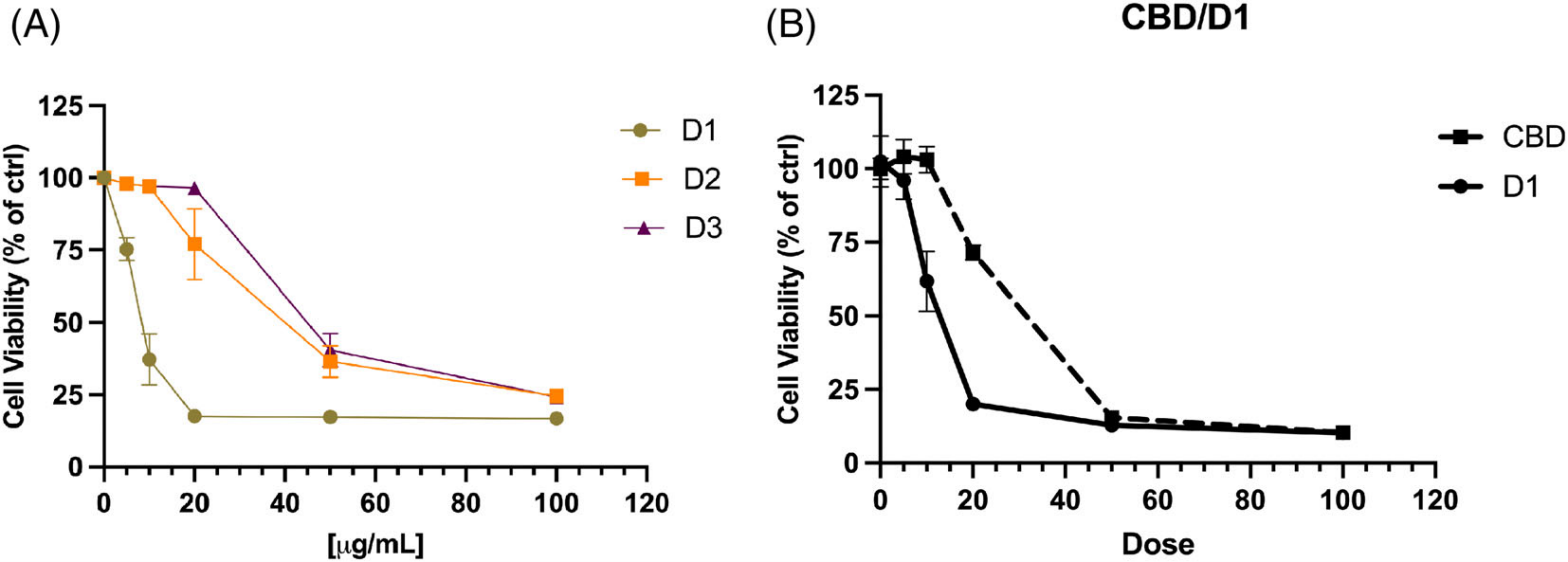
(a) Dose/response curves of decarboxylated D1, D2 and D3 hemp extracts at 5–100 μg/ml on K562 cells after 48 hr of treatments. Data in the graphs are shown as mean ± SD (*n* = 4 per group). (b) Effect of pure CBD on K562 cells after 48 hr of treatment compared with the extract D1. The dose is shown as μg/ml for D1 extract and μM for CBD, respectively. Data in the graphs are shown as mean ± SD (*n* = 4 per group)

In addition to the D1 extract, others (D2 and D3) having a different cannabinoid profile were tested for their ability to modulate the K562 cell viability after 48 hr of incubation time. As shown in Figure 3a, D1 demonstrated to be more powerful than D2 and D3. Indeed, D1 reached an IC_50_ of 11.6 μg/ml, whereas D2 and D3 showed an IC_50_ of 40.9 and 66.7 μg/ml respectively. The effect of D1 on K562 cell viability was time‐dependent, reaching the maximal effect after 72 hr of incubation time with an IC_50_ of 9.8 μg/ml (Figure S2).

Furthermore, the activity of the D1 extract was assessed on human peripheral blood mononuclear cells (PBMC) to exclude a cytotoxic effect on non‐cancer cells. To do this, we used a concentration of D1 extract higher than the IC_50_ found on K562 cells, in order to increase the probability of a cytotoxic event on PBMCs. As shown in Figure S3, D1 did not affect the cell viability of PBMCs after 48 hr treatment at 50 μg/ml, indicating that D1 activity did not interfere with cell viability of normal hematopoietic cells.

Since D1 was rich in CBD, the activity of pure CBD on K562 cells was assessed. As reported in Figure 3b, D1 showed a lower IC_50_ compared with pure CBD. Indeed, CBD was able to decrease 50% of cell viability at a concentration of 28 μM, whereas the IC_50_ of D1 was 11.6 μg/ml, corresponding to 16 μM in CBD‐equivalents. The equivalence was calculated considering that CBD was quantified in D1 extract at 440.8 mg/g (Table 2). This difference could be explained considering the chemical composition complexity of the extract. Even if CBD is the main component to which the activity of the extract could be ascribed, other minor compounds can work in a synergic way to enhance the overall antiproliferative activity of D1.

### 3.4 Identification of CBD and related oxidation products in K562 cell lysates

K562 cell lysates treated with D1 were analysed in UHPLC‐HRMS in order to identify compounds related to CBD that can be formed under oxidative conditions. Indeed, CBD, in the presence of oxygen, may be oxidized to monomeric and dimeric hydroxyquinones (Mechoulam & Hanus, 2002). The monomeric CBD‐hydroxyquinone is known to have a high antiproliferative activity (Wilson, Fief, Jackson, Mercer, & Deweese, 2018). In the attempt to investigate the presence of these compounds in cell lysates, a target HRMS analysis was used using MS data of CBD oxidation products previously described in the literature (Mechoulam & Hanus, 2002) (Table S2).

Only CBD was found in the D1‐treated cell lysates, both in the positive and in the negative ion mode, at a retention time of 16.7 min. None of the compounds derived from CBD oxidation were detected in cell culture medium RPMI and in the lysates from both treated and non‐treated cells. These findings suggest that the antiproliferative activity of the D1 extract cannot be attributed to the formation of CBD oxidized derivatives, such as monomeric and dimeric hydroxyquinones.

### 3.5 Effect of the CBD‐rich hemp extract on K562 cell cycle

**FIGURE 4:**
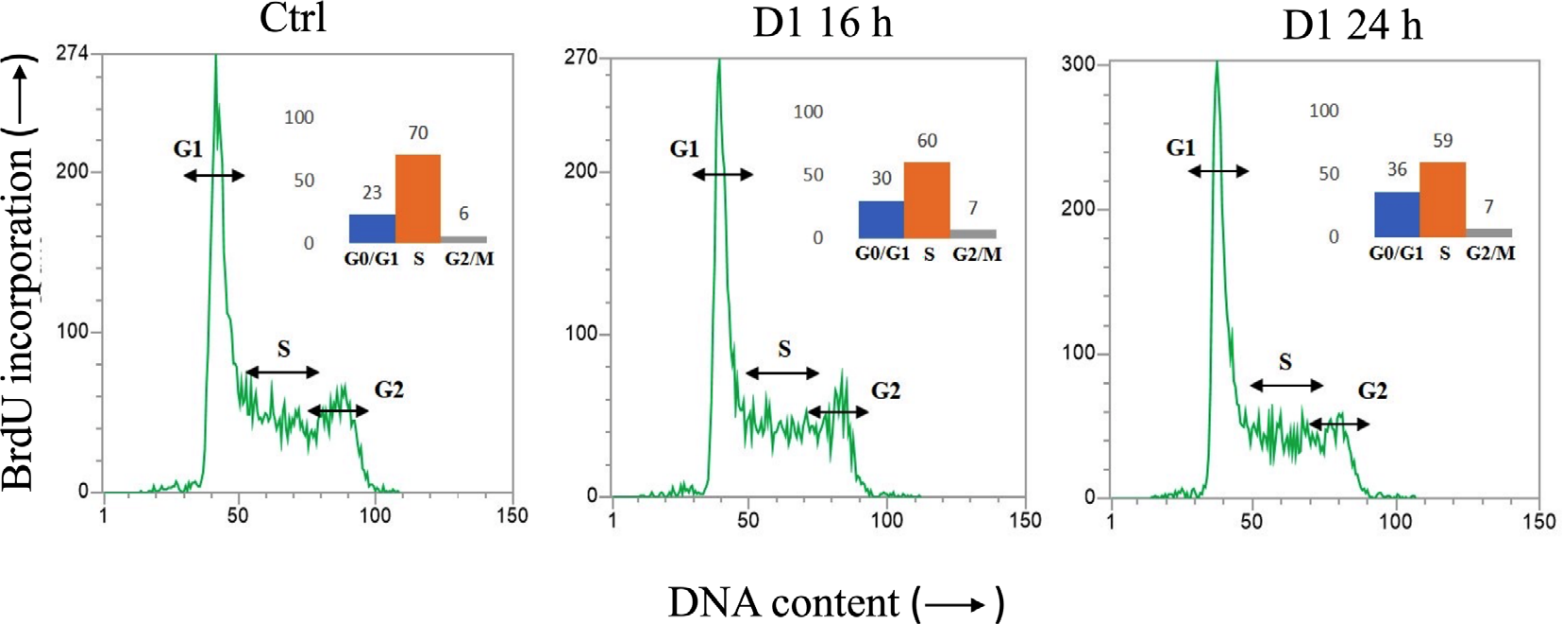
Cell cycle profile and percentage of cells in G0/G1, S and/or G2/M phase

To understand the possible molecular mechanism exerted by D1, K562 cell cycle distribution was further evaluated to investigate whether the extract was able to cause cell cycle arrest at different time points. A concentration of 20 μg/ml was selected from the previous experiments on cell viability. The flow cytometry analysis revealed that K562 cell cycle was not affected by the treatment with the CBD‐rich decarboxylated extract D1. Indeed, D1 did not change cell cycle parameters both at 16 and 24 hr of incubation time (Figure 4), indicating that the activity of the extract did not involve the modulation of K562 cell cycle.

### 3.6 Effect of the CBD‐rich hemp extract on apoptosis of K562 cells

**FIGURE 5:**
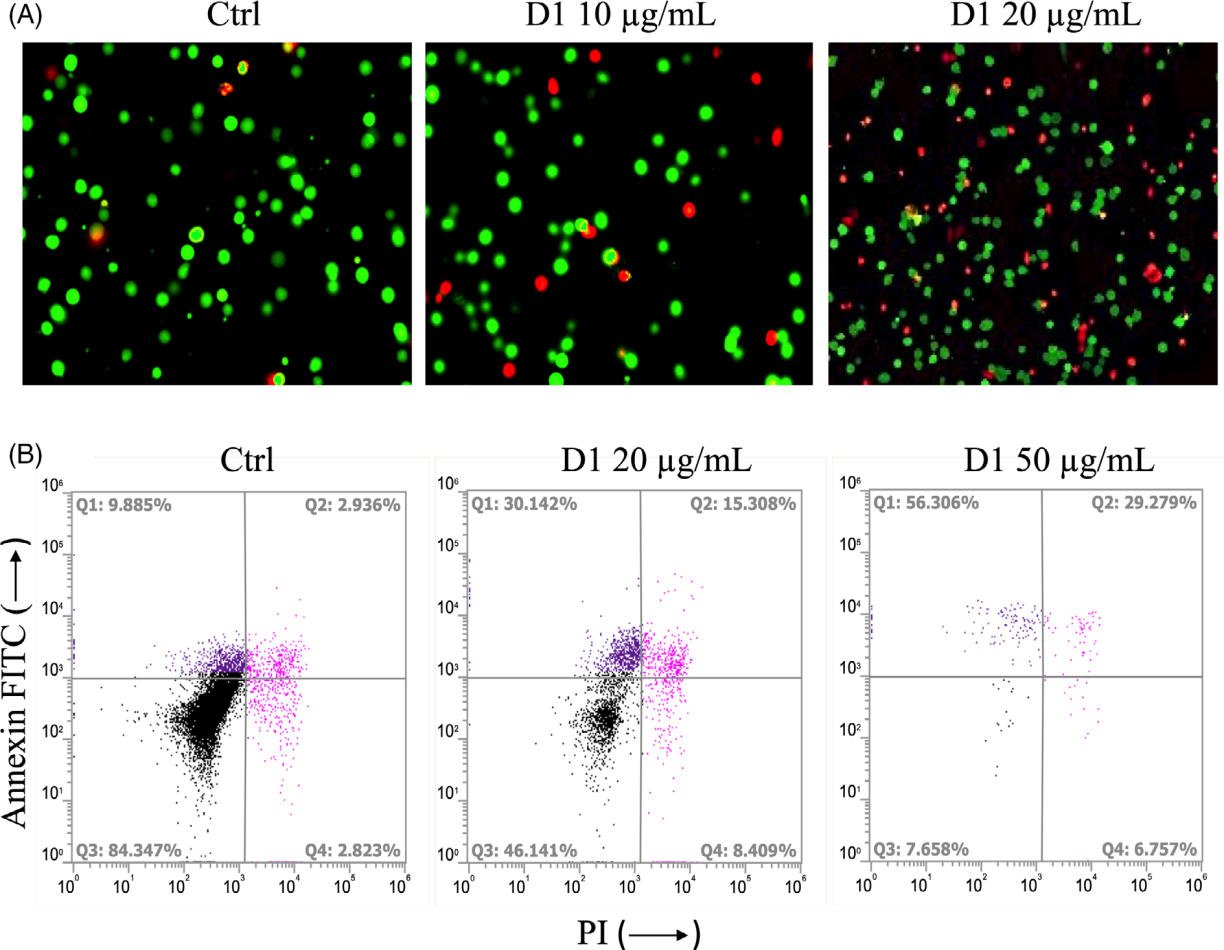
(a) Fluorescence microscopy analysis of K562 cells treated with 10 and 20 μg/ml of the D1 extract at 16 hr and stained with calcein (green dots) and PI (red dots). (b) Flow cytometric analysis of K562 cells stained with Annexin‐V and propidium iodide (PI). Representative dot plots of K562 cells after a 16 hr treatment with increasing concentrations of D1. (Q1) Cells undergoing apoptosis. (Q2, Q4) Late apoptosis or necrosis. (Q3) Living cells without signs of apoptosis

The previous results led us to focus our attention on cell death induced by D1. The ability of D1 to induce either the apoptotic or necrotic pathway in K562 cells after an overnight treatment at different concentrations was evaluated. Treated and non‐treated K562 cells were stained with calcein and propidium iodide to discriminate through fluorescence microscopy the population of living cells (green spots) in opposition to the necrotic ones (red spots). After 16 hr of treatment at 10 μg/ml of the D1 extract, there was an increase in PI related‐necrotic cells (Figure 5a), which became more remarkable in cells treated with 20 μg/ml of D1. Moreover, the treatment of K562 with D1 at 20 μg/ml elicited a significant increase of apoptosis in comparison to control, as shown by Annexin V fluorescence accumulation (Figure 5b). In particular, the percentage of cells stained with Annexin V were increased from 9.8% in the control group to 30.1% in the cells treated with D1 at 20 μg/ml. The percentage of double stained cells, that represents the late stage of apoptosis/necrosis, changed from 2.9% in the control group to 15.3% after the treatment with D1 at 20 μg/ml. The treatment at 50 μg/ml caused the death of almost all the cell population, making cell count difficult.

### 3.7 Effect of the CBD‐rich hemp extract on the expression of proteins involved in apoptosis and on cytochrome‐c release

**FIGURE 6:**
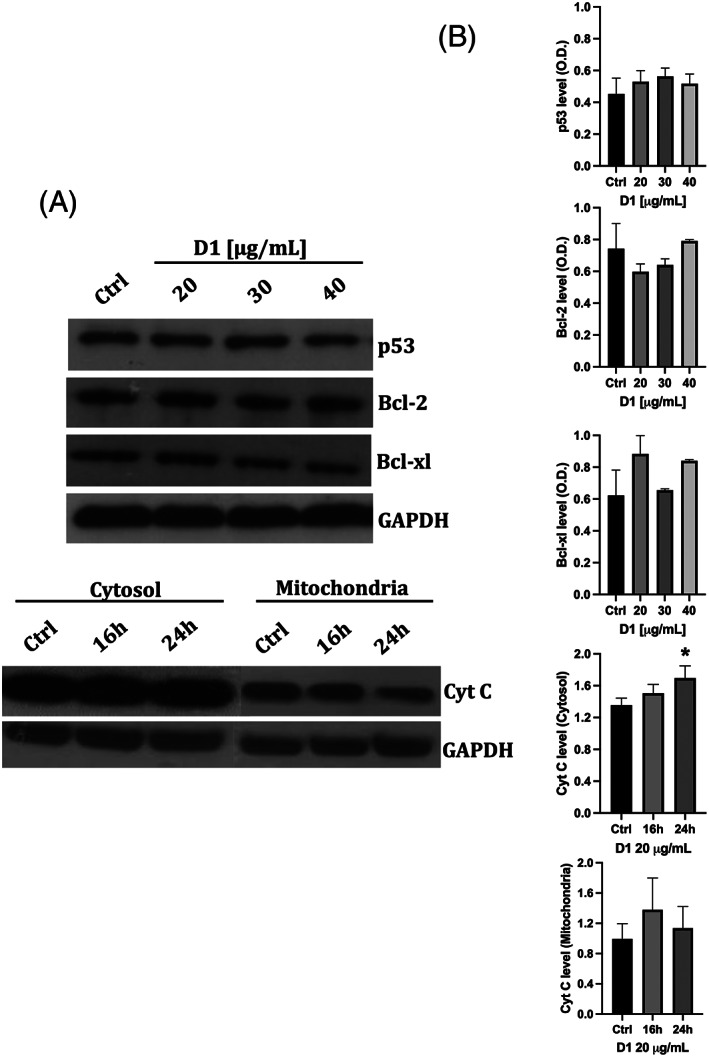
Effect of the D1 extract on p53, bcl‐2, bcl‐xl protein expression in K562 cells and on cytochrome c release. (a) Representative western blots at different concentration/time treatments. (b) Densitometric analyses of protein levels of p53, bcl‐2, and bcl‐xl of K562 cells lysate after incubation with 20, 30 and 40 μg/ml of D1 for 16 hr; densitometric analyses of cytosolic and mitochondrial cytochrome c in K562 cells lysate after 16 and 24 hr incubation with 20 μg/ml of D1. Densitometry values were normalized to the protein loading control GAPDH. The values are expressed as mean ± SD of three independent experiments (*n* = 3 per group)

To get more insights on cell death in leukaemia K562 cells, the expression and activity of apoptosis‐related proteins, such as p53, Bcl‐2 and Bcl‐xl, was evaluated using 20, 30 and 40 μg/ml of D1 after a 16 hr treatment. As shown in Figure 6, the treatment with D1 did not affect the protein expression of p53, Bcl‐2 and Bcl‐xl at concentrations that induced apoptosis in previous experiments with this cell line. These findings suggest that apoptosis in K562 cells is not mediated neither by the pro‐apoptotic marker p53 nor by the anti‐apoptotic markers Bcl‐2 and Bcl‐xl.

The release of cytochrome‐c into the cytoplasm is another hallmark of apoptosis. The measurement of cytochrome‐c following 16 and 24 hr treatment with the D1 extract at 20 μg/ml produced a significant increase in the cytochrome‐c content in the cytoplasm compared to the control only after 24 hr (Figure 6b), whereas the difference in the mitochondrial fraction was not statistically significant.

### 3.8 Effect of the CBD‐rich hemp extract on caspase 3 and 7

**FIGURE 7:**
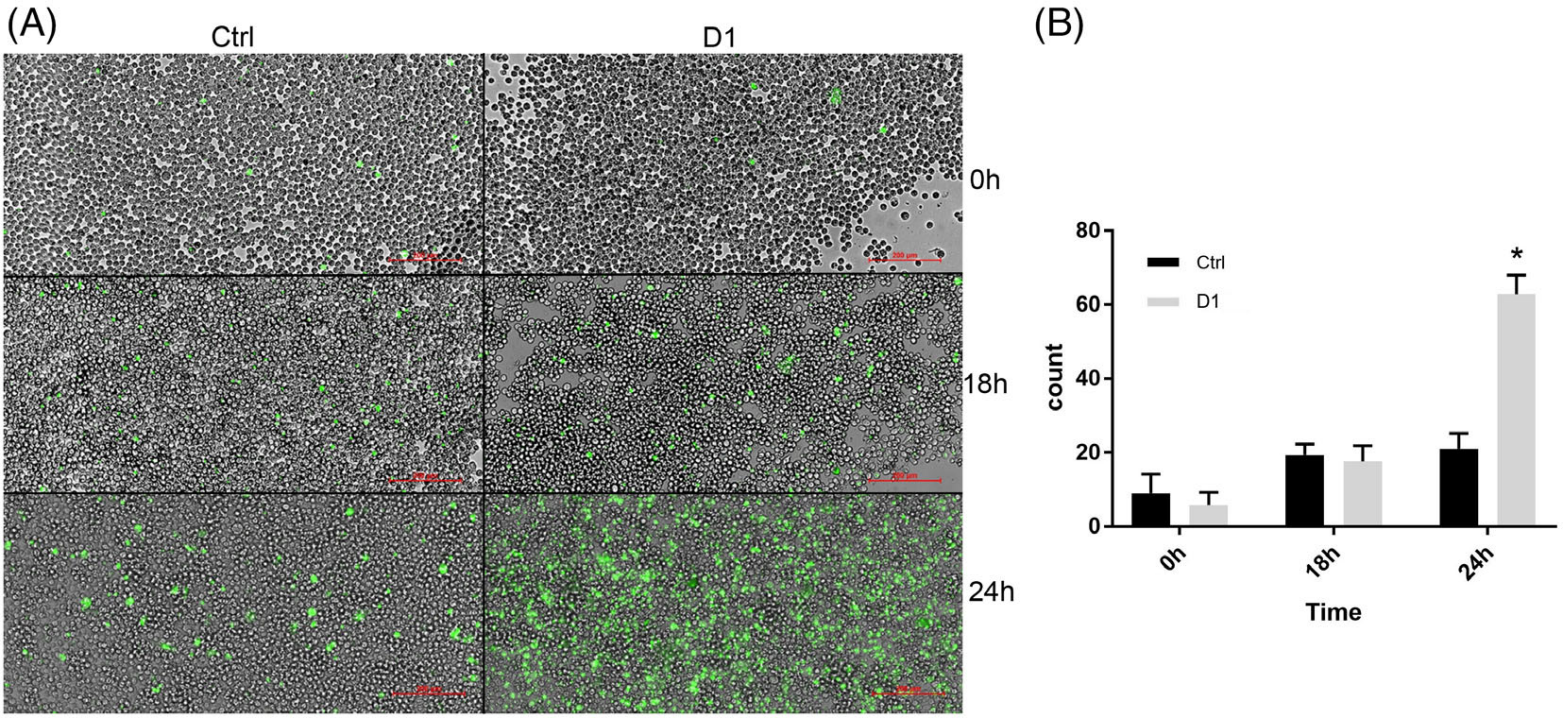
(a) Live imaging microphotographs taken with a 20× objective. Caspase – activated cells (green) shown at different time points (0, 18 and 24 hr) in the control (Ctrl) and in the cells treated with D1. Scale bar (red) 200 μm. (b) Histogram of caspase – activated positive cells for each condition, *p < 0.05 compared to control. For all panels, data are expressed as mean ± SD (*n* = 4 per group)

To assess the number of caspase – activated cells after the treatment with D1, a live imaging kinetic assay through 48 hr observation was performed. The numbers of positive cells remained relatively similar at least for the first 18 hr (Figure 7a). After this time point, the count of caspase – activated cells of the samples treated with D1 rapidly increased, reaching a peak at around 24 hr, as shown in the related panel of Figure 7a. Compared with the control, this difference was statistically significant (Figure 7b).

It is important to note that, while in the first 18 hr caspase activation between treated and untreated samples did not differ, the total number of cells in the wells were not similar in comparison. The area covered with cells steadily increased in control condition during the observation, while this did not happen in D1 treated cells. After 18 hr, when caspase activation became statistically significant, this effect on total cell growth was further magnified.

Taken together, these data suggest that the effect on the decrease of cell population in D1 treated cells was not, for the first 18 hr, related to the effect of caspase 3/7 activation which occurs later, but rather on the alteration of mitochondrial membrane potential (ΔΨm) itself. However, after 18 hr, apoptosis was a predominant mechanism that contributed to massive cell death, possibly enhanced by cell death signal cumulated in the previous hours.

### 3.9 Effect of the CBD‐rich hemp extract on the mitochondrial membrane potential

It is well known that the mitochondrial membrane potential decreases during apoptosis and matrix remodelling is required to attain complete cytochrome c release from mitochondria (Gottlieb, Armour, Harris, & Thompson, 2003).

To evaluate the possible effect on mitochondria in D1 treated cells, we used JC1 staining and followed the fluorescence levels from 0 to 24 hr after treatment.

**FIGURE 8:**
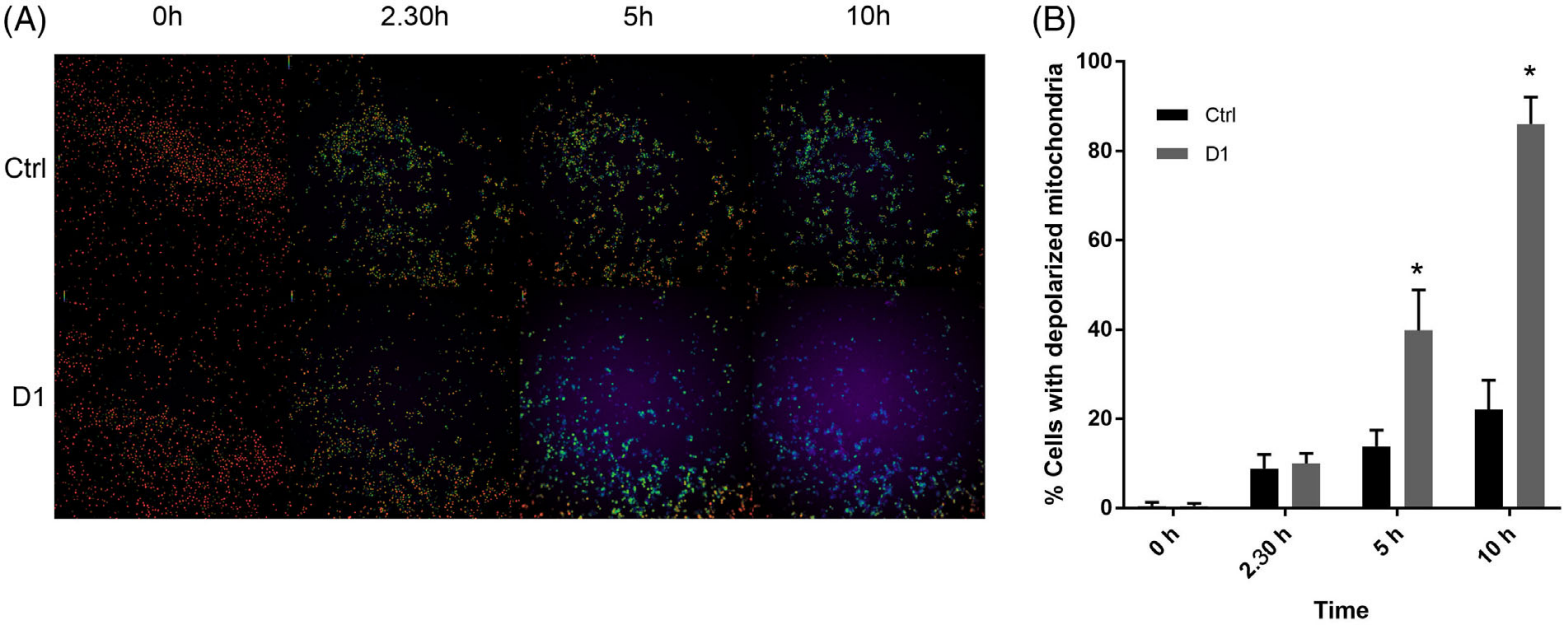
(a) Live imaging microphotographs taken with a 10x objective. Ratiometric view ranging from violet (totally depolarized mitochondria) to bright green (polarized mitochondria) shown at different time points (0, 2.30, 5 and 10 hr) in the control (Ctrl) and in the cells treated with D1 cells. Each image composing the panel shows an area of 1770 μm × 1770 μm. (b) Histogram showing the number of polarized mitochondria positive cells for each condition at 0, 2.30, 5 and 10 hr, *p <  0.05 compared to control. For all panels, data are expressed as mean ± SD (*n* = 6 per group)

Starting from 5 hr of treatment, D1 entailed a drop in mitochondrial membrane potential (Figure 8a): the number of cells showing no polarized mitochondria at all (ratio level under 1.5 in a scale from 0 to 4.5), a sign of mitochondrial distress, raised in a significant way. At 10 hr, the effects were clearly visible. These data justify the count of apoptotic cells observed in the caspase assay at 24 hr, that is, cell death occurs after mitochondrial failure.

### 3.10 Effect of the CBD‐rich hemp extract on mechanical properties of individual K562 cells

By using the micropipette aspiration technique (MAT), the mechanical properties of individual K562 cells in the control situation and exposed to D1 at 30 μg/ml were evaluated. Mechanical characterization by MAT can explore aspects of active reorganization of cells. For example, it has been shown that myosin II can accumulate in the part of the cell inside the micropipette during an aspiration experiment and it can induce a retraction of the cell at constant applied pressure (Mohan, Luo, Robinson, & Iglesias, 2015). The process, due to the catch bond character of the myosin II/actin interaction, leads to an oscillating behaviour of the cell projection inside the micropipette at constant applied pressure. This type of behaviour has been associated also to the first stages of the apoptotic process, in which the activity of the myosin II motor is specifically increased (Desouza, Gunning, & Stehn, 2012). In this case, the behaviour of K562 cells in the control conditions (culture medium) and when the cells were exposed to 30 μg/ml of D1 extract for 18 hr were compared. Whereas in the case of control a continuous growth of the protrusion was observed for all the tested cells inside the micropipette after the pressure step was applied (*n* = 20), in the case of the exposure to D1 6 cells out of 20 showed an oscillatory behaviour. Figure S4 shows representative traces for the two cases. This result suggests an induction of apoptotic processes elicited by the D1 treatment in cells that under the optical microscope showed no particular difference with respect to control cells, thus confirming the data obtained on mitochondrial membrane potential and caspase activation.

## 4 CONCLUSIONS

The present work was aimed at the chemical characterization of different hemp extracts and at the assessment of their antiproliferative activity. Cancer cell lines of different embryological origin were used as in vitro models and among these the chronic myelogenous leukaemia cell line K562 was the most sensitive to the treatment with hemp extracts. The decarboxylated extract D1, rich in CBD, was able to affect the cell viability of K562 at a concentration of 11.6 μg/ml after 48 hr. Compared with pure CBD, the extract D1 elicited a higher antiproliferative activity, suggesting a synergistic effect, that may be due to the presence of other minor compounds in the extract, even if CBD was the most abundant one. Interestingly, the D1 extract was unable to interfere with the viability of PBMCs, indicating a very low level of toxicity in vitro at the concentration tested.

The mechanism of K562 cell death was investigated and cytofluorimetric analysis showed a time and concentration‐dependent increase in apoptosis after D1 treatment.

Apoptosis was attributed to the activation of caspase 3 and 7, but it did not involve the main pro‐ and anti‐apoptotic pathways, such as p53, Bcl‐2 and Bcl‐xl. CBD is known to directly modulate the voltage‐dependent ion channel 1 (VDAC1), resulting in reduced channel conductance and, eventually, to cell death. Since we reported for D1 an increase in the cytochrome c levels in the cytosol together with the modulation of the mitochondrial membrane potential and activation of caspase 3 and 7, we believe that the K562 cell death mediated by D1 might be related to the activity of the CBD‐rich extract on VDAC1, rather than a direct modulation of the Bcl‐2 family. However, it must be remarked that we tested only two of the major players involved in the induction of the apoptotic processes, and the involvement of some other Bcl2‐related family members, such as Bax or the BH3‐only protein Bim, cannot be excluded.

Moreover, further investigation by proteomics will be necessary to unveil the specific molecular targets of CBD‐rich hemp extracts, focusing on alternative pathways, which differ from the canonical CB_1_, CB_2_, TRPV1 and GPR55 receptors.

Even if further work is necessary to validate the results on different cell lines, the present research supports D1 and, therefore, its main component CBD as a possible candidate for future therapy of myeloproliferative diseases either alone or in association with other anticancer drugs.

## Supporting information


**Data S1.** Supporting information.

## Data Availability

The data that supports the findings of this study are available in the supplementary material of this article.
