# Supplementary material for: Chemical characterization of non‐psychoactive Cannabis sativa L. extracts, in vitro antiproliferative activity and induction of apoptosis in chronic myelogenous leukaemia cancer cells
Source: Phytother Res. 2022 Feb 2;36(2):914–27. doi: 10.1002/ptr.7357 (PMC9304126; doi:10.1002/ptr.7357)
Supplement: Supplementary file 1 — Data S1. Supporting information. [file PTR-36-914-s001.docx]

**Supporting Information for Publication**

Lisa Anceschi ^1,2^, Alessandro Codeluppi ^1^, Virginia Brighenti ^1^, Riccardo Tassinari ^3^, Valentina Taglioli ^4^, Lucia Marchetti ^1,2^, Luca Roncati ^5^, Andrea Alessandrini ^6,7,8^, Lorenzo Corsi ^1,8*^, Federica Pellati ^1,*^

*^1^ Department of Life Sciences, University of Modena and Reggio Emilia, Via G. Campi 103-287, 41125 Modena, Italy*

*^2^ Clinical and Experimental Medicine PhD Program, University of Modena and Reggio Emilia, Via G. Campi 287, 41125 Modena, Italy*

*^3^ National Laboratory of Molecular Biology and Stem Cell Bioengineering-Eldor Lab, National Institute of Biostructures and Biosystems (NIBB), Innovation Accelerator, CNR, Via P. Gobetti 101, 40129 Bologna, Italy*

*^4^ Department of Experimental, Diagnostic and Specialty Medicine (DIMES), Alma Mater Studiorum University of Bologna, Via Massarenti 9, 40138 Bologna, Italy*

*^5^ Institute of Pathology, University of Modena and Reggio Emilia, Via del Pozzo 71, 41125 Modena, Italy*

*^6^ Department of Physics, Informatics and Mathematics, University of Modena and Reggio Emilia, Via G. Campi 213/a, 41125 Modena, Italy*

*^7^ CNR-Nanoscience Institute-S3, Via G. Campi 213/a, 41125 Modena, Italy*

*^8^ Istituto Nazionale di Biostrutture e Biosistemi (INBB), Viale Medaglie d’Oro 305, 00136 Roma, Italy*

*Corresponding authors:

Prof. Federica Pellati (medicinal chemistry section)

Phone +39 059 2058565

E-mail: [federica.pellati@unimore.it](mailto:federica.pellati@unimore.it)

Prof. Lorenzo Corsi (pharmacology section)

Phone +39 059 2055376

E-mail: [lorenzo.corsi@unimore.it](mailto:lorenzo.corsi@unimore.it)

| Table S1. List of potential masses of neutral and acid cannabinoids in target UHPLC-HRMS metabolomic analysis of hemp extracts. | | | | | |
| --- | --- | --- | --- | --- | --- |
| NEUTRAL CANNABINOIDS | | | **ACID CANNABINOIDS** | | |
| Compound | Formula | Molecular weight | Compound | Formula | Molecular weight |
| Cannabiorcol-C1 (CBNO) | C₁₇H₁₈O₂ | 254,12340 | CBNOA | C₁₈H₁₈O₄ | 298,11323 |
| CBNDO | C₁₇H₁₈O₂ | 254,12340 | CBNDOA | C₁₈H₁₈O₄ | 298,11323 |
| Δ⁹-THCO | C₁₇H₂₂O₂ | 258,15470 | Δ⁹-THCOA | C₁₈H₂₂O₄ | 302,14453 |
| CBDO | C₁₇H₂₂O₂ | 258,15470 | CBDOA | C₁₈H₂₂O₄ | 302,14453 |
| CBCO | C₁₇H₂₂O₂ | 258,15470 | CBCOA | C₁₈H₂₂O₄ | 302,14453 |
| Δ⁸-THCO | C₁₇H₂₂O₂ | 258,15470 | Δ⁸-THCOA | C₁₈H₂₂O₄ | 302,14453 |
| CBLO | C₁₇H₂₂O₂ | 258,15470 | CBLOA | C₁₈H₂₂O₄ | 302,14453 |
| CBGO | C₁₇H₂₄O₂ | 260,17035 | CBGOA | C₁₈H₂₄O₄ | 304,16018 |
| CBN-C2 | C₁₈H₂₀O₂ | 268,13905 | CBNA-C2 | C₁₉H₂₀O₄ | 312,12888 |
| CBND-C2 | C₁₈H₂₀O₂ | 268,13905 | CBNDA-C2 | C₁₉H₂₀O₄ | 312,12888 |
| Δ⁹-THC-C2 | C₁₈H₂₄O₂ | 272,17035 | Δ⁹-THCA-C2 | C₁₉H₂₄O₄ | 316,16018 |
| CBD-C2 | C₁₈H₂₄O₂ | 272,17035 | CBDA-C2 | C₁₉H₂₄O₄ | 316,16018 |
| CBC-C2 | C₁₈H₂₄O₂ | 272,17035 | CBCA-C2 | C₁₉H₂₄O₄ | 316,16018 |
| Δ⁸-THC-C2 | C₁₈H₂₄O₂ | 272,17035 | Δ⁸-THCA-C2 | C₁₉H₂₄O₄ | 316,16018 |
| CBL-C2 | C₁₈H₂₄O₂ | 272,17035 | CBLA-C2 | C₁₉H₂₄O₄ | 316,16018 |
| CBEO | C₁₇H₂₂O₃ | 274,14962 | CBEOA | C₁₈H₂₂O₅ | 318,13945 |
| CBG-C2 | C₁₈H₂₆O₂ | 274,18600 | CBGA-C2 | C₁₉H₂₆O₄ | 318,17583 |
| CBNV | C₁₉H₂₂O₂ | 282,15470 | CBNVA | C₂₀H₂₂O₄ | 326,14453 |
| CBNDV | C₁₉H₂₂O₂ | 282,15470 | CBNDVA | C₂₀H₂₂O₄ | 326,14453 |
| Δ⁹-THCV | C₁₉H₂₆O₂ | 286,18600 | Δ⁹-THCVA | C₂₀H₂₆O₄ | 330,17583 |
| CBDV | C₁₉H₂₆O₂ | 286,18600 | CBDVA | C₂₀H₂₆O₄ | 330,17583 |
| CBCV | C₁₉H₂₆O₂ | 286,18600 | CBCVA | C₂₀H₂₆O₄ | 330,17583 |
| Δ⁸-THCV | C₁₉H₂₆O₂ | 286,18600 | Δ⁸-THCVA | C₂₀H₂₆O₄ | 330,17583 |
| CBLV | C₁₉H₂₆O₂ | 286,18600 | CBLVA | C₂₀H₂₆O₄ | 330,17583 |
| 2-Methyl-2-(4-methyl-2-pentenyl)-7-propyl-2H-1-benzopyran-5-ol | C₁₉H₂₆O₂ | 286,18600 | 2-Methyl-2-(4-methyl-2-pentenyl)-7-propyl-2H-1-benzopyran-5-ol acid | C₂₀H₂₆O₄ | 330,17583 |
| Δ⁷-THCV | C₁₉H₂₆O₂ | 286,18600 | Δ⁷-THCVA | C₂₀H₂₆O₄ | 330,17583 |
| CBE-C2 | C₁₈H₂₄O₃ | 288,16527 | CBEA-C2 | C₁₉H₂₄O₅ | 332,15510 |
| CBGV | C₁₉H₂₈O₂ | 288,20165 | CBGVA | C₂₀H₂₈O₄ | 332,19148 |
| CBTO | C₁₇H₂₂O₄ | 290,14453 | CBTOA | C₁₈H₂₂O₆ | 334,13436 |
| CBN-C4 | C₂₀H₂₄O₂ | 296,17035 | CBNA-C4 | C₂₁H₂₄O₄ | 340,16018 |
| CBND-C4 | C₂₀H₂₄O₂ | 296,17035 | CBNDA-C4 | C₂₁H₂₄O₄ | 340,16018 |
| Δ⁹-THC-C4 | C₂₀H₂₈O₂ | 300,20165 | Δ⁹-THCA-C4 | C₂₁H₂₈O₄ | 344,19148 |
| CBD-C4 | C₂₀H₂₈O₂ | 300,20165 | CBDA-C4 | C₂₁H₂₈O₄ | 344,19148 |
| CBC-C4 | C₂₀H₂₈O₂ | 300,20165 | CBCA-C4 | C₂₁H₂₈O₄ | 344,19148 |
| (-)-*trans*-Δ⁸-THC-C4 | C₂₀H₂₈O₂ | 300,20165 | (-)-*trans*-Δ⁸-THCA-C4 | C₂₁H₂₈O₄ | 344,19148 |
| CBL-C4 | C₂₀H₂₈O₂ | 300,20165 | CBLA-C4 | C₂₁H₂₈O₄ | 344,19148 |
| CBEV | C₁₉H₂₆O₃ | 302,18092 | CBEVA | C₂₀H₂₆O₅ | 346,17075 |
| CBG-C4 | C₂₀H₃₀O₂ | 302,21730 | CBGA-C4 | C₂₁H₃₀O₄ | 346,20713 |
| CBT-C2 | C₁₈H₂₄O₄ | 304,16018 | CBTA-C2 | C₁₉H₂₄O₆ | 348,15001 |
| Cannabichromanone-C3 | C₁₈H₂₄O₄ | 304,16018 | Cannabichromanonic acid-C3 | C₁₉H₂₄O₆ | 348,15001 |
| Cannabiglendol-C3 | C₁₉H₂₈O₃ | 304,19657 | Cannabiglendolic acid-C3 | C₂₀H₂₈O₅ | 348,1864 |
| CBX | C₂₁H₂₂O₂ | 306,15470 | CBXA | C₂₂H₂₂O₄ | 350,14453 |
| DCBF | C₂₁H₂₄O₂ | 308,17035 | DCBFA | C₂₂H₂₄O₄ | 352,16018 |
| CBN | C₂₁H₂₆O₂ | 310,18600 | CBNA | C₂₂H₂₆O₄ | 354,17583 |
| CBND | C₂₁H₂₆O₂ | 310,18600 | CBNDA | C₂₂H₂₆O₄ | 354,17583 |
| CBF | C₂₁H₂₆O₂ | 310,18600 | CBFA | C₂₂H₂₆O₄ | 354,17583 |
| Δ⁹-THC | C₂₁H₃₀O₂ | 314,21730 | Δ⁹-THCA | C₂₂H₃₀O₄ | 358,20713 |
| Δ⁸-THC | C₂₁H₃₀O₂ | 314,21730 | Δ⁸-THCA | C₂₂H₃₀O₄ | 358,20713 |
| CBC | C₂₁H₃₀O₂ | 314,21730 | CBCA | C₂₂H₃₀O₄ | 358,20713 |
| CBD | C₂₁H₃₀O₂ | 314,21730 | CBDA | C₂₂H₃₀O₄ | 358,20713 |
| CBL | C₂₁H₃₀O₂ | 314,21730 | CBLA | C₂₂H₃₀O₄ | 358,20713 |
| CBR | C₂₁H₃₀O₂ | 314,21730 | CBRA | C₂₂H₃₀O₄ | 358,20713 |
| (-)-*cis*-Δ⁹-THC | C₂₁H₃₀O₂ | 314,21730 | (-)-cis-Δ⁹-THCA | C₂₂H₃₀O₄ | 358,20713 |
| *trans*-iso-Δ⁷-THC | C₂₁H₃₀O₂ | 314,21730 | trans-iso-Δ⁷-THCA | C₂₂H₃₀O₄ | 358,20713 |
| CBE-C4 | C₂₀H₂₈O₃ | 316,19657 | CBEA-C4 | C₂₁H₂₈O₅ | 360,18640 |
| CBG | C₂₁H₃₂O₂ | 316,23295 | CBGA | C₂₂H₃₂O₄ | 360,22278 |
| CBTV | C₁₉H₂₆O₄ | 318,17583 | CBTVA | C₂₀H₂₆O₆ | 362,16566 |
| CBNM | C₂₂H₂₈O₂ | 324,20165 | CBNMA | C₂₃H₂₈O₄ | 368,19148 |
| CBNDM-C5 | C₂₂H₂₈O₂ | 324,20165 | CBNDMA-C5 | C₂₃H₂₈O₄ | 368,19148 |
| OH-CBN | C₂₁H₂₆O₃ | 326,18092 | OH-CBNA | C₂₂H₂₆O₅ | 370,17075 |
| OH-CBND | C₂₁H₂₆O₃ | 326,18092 | OH-CBNDA | C₂₂H₂₆O₅ | 370,17075 |
| OTHC | C₂₁H₂₈O₃ | 328,19657 | OTHCA | C₂₂H₂₈O₅ | 372,1864 |
| Cannabichromanone D-C5 | C₂₁H₂₈O₃ | 328,19657 | Cannabichromanonic acid D-C5 | C₂₂H₂₈O₅ | 372,1864 |
| CBCON-C5 | C₂₁H₂₈O₃ | 328,19657 | CBCONA | C₂₂H₂₈O₅ | 372,1864 |
| CBDM | C₂₂H₃₂O₂ | 328,23295 | CBDMA | C₂₃H₃₂O₄ | 372,22278 |
| Δ⁹-THCM-C5 | C₂₂H₃₂O₂ | 328,23295 | Δ⁹-THCMA | C₂₃H₃₂O₄ | 372,22278 |
| (±)-3''-hydroxy-Δ⁴''-cannabichromene-C5 | C₂₁H₃₀O₃ | 330,21222 | (±)-3''-hydroxy-Δ⁴''-cannabichromenic acid-C5 | C₂₂H₃₀O₅ | 374,20205 |
| CBE | C₂₁H₃₀O₃ | 330,21222 | CBEA | C₂₂H₃₀O₅ | 374,20205 |
| 2-geranyl-5-hydroxy-3-n-pentyl-1,4-benzoquinone-C5 | C₂₁H₃₀O₃ | 330,21222 | 2-geranyl-5-hydroxy-3-n-pentyl-1,4-benzoquinonic acid-C5 | C₂₂H₃₀O₅ | 374,20205 |
| 8α-OH-Δ⁹-THC | C₂₁H₃₀O₃ | 330,21222 | 8α-OH-Δ⁹-THCA | C₂₂H₃₀O₅ | 374,20205 |
| 8β-OH-Δ⁹-THC | C₂₁H₃₀O₃ | 330,21222 | 8β-OH-Δ⁹-THCA | C₂₂H₃₀O₅ | 374,20205 |
| 10α-OH-Δ⁸-THC | C₂₁H₃₀O₃ | 330,21222 | 10α-OH-Δ⁸-THCA | C₂₂H₃₀O₅ | 374,20205 |
| 10β-OH-Δ⁸-THC | C₂₁H₃₀O₃ | 330,21222 | 10β-OH-Δ⁸-THCA | C₂₂H₃₀O₅ | 374,20205 |
| 10α-hydroxy-Δ⁹-hexahydrocannabinol-C5 | C₂₁H₃₀O₃ | 330,21222 | 10α-hydroxy-Δ⁹-hexahydrocannabinolic acid-C5 | C₂₂H₃₀O₅ | 374,20205 |
| 9β,10β-Epoxyhexahydrocannabinol-C5 | C₂₁H₃₀O₃ | 330,21222 | 9β,10β-Epoxyhexahydrocannabinolic acid-C5 | C₂₂H₃₀O₅ | 374,20205 |
| OH-CBD | C₂₁H₃₀O₃ | 330,21222 | OH-CBDA | C₂₂H₃₀O₅ | 374,20205 |
| CBGM | C₂₂H₃₄O₂ | 330,24860 | CBGMA | C₂₃H₃₄O₄ | 374,23843 |
| Cannabichromanone-C5 | C₂₀H₂₈O₄ | 332,19148 | Cannabichromanonic acid-C5 | C₂₁H₂₈O₆ | 376,18131 |
| CBT-C4 | C₂₀H₂₈O₄ | 332,19148 | CBTA-C4 | C₂₁H₂₈O₆ | 376,18131 |
| (±)-6,7-*cis*-epoxycannabigerol-C5 | C₂₁H₃₂O₃ | 332,22787 | (±)-6,7-*trans*-epoxycannabigerolic acid-C5 | C₂₂H₃₂O₅ | 376,21770 |
| (±)-6,7-*trans*-epoxycannabigerol-C5 | C₂₁H₃₂O₃ | 332,22787 | (±)-6,7-*cis*-epoxycannabigerolic acid-C5 | C₂₂H₃₂O₅ | 376,21770 |
| (-)-7-hydroxycannabichromane-C5 | C₂₁H₃₂O₃ | 332,22787 | (-)-7-hydroxycannabichromanic acid-C5 | C₂₂H₃₂O₅ | 376,21770 |
| Cannabimovone-C5 | C₂₁H₃₀O₄ | 346,20713 | Cannabimovonic acid-C5 | C₂₂H₃₀O₆ | 390,19696 |
| (-)-*trans*-CBT | C₂₁H₃₀O₄ | 346,20713 | (-)-*trans*-CBTA | C₂₂H₃₀O₆ | 390,19696 |
| (+)-*trans*-CBT | C₂₁H₃₀O₄ | 346,20713 | (+)-*trans*-CBTA | C₂₂H₃₀O₆ | 390,19696 |
| (±)-cis-CBT | C₂₁H₃₀O₄ | 346,20713 | (±)-*cis*-CBTA | C₂₂H₃₀O₆ | 390,19696 |
| (-)-*trans*-CBT-OEt | C₂₁H₃₀O₄ | 346,20713 | (-)-*trans*-CBTA-OEt | C₂₂H₃₀O₆ | 390,19696 |
| CBR | C₂₁H₃₂O₄ | 348,22278 | CBRA | C₂₂H₃₂O₆ | 392,21261 |
| Cannabichromanone C-C5 | C₂₁H₂₈O₅ | 360,18640 | Cannabichromanonic acid C-C5 | C₂₂H₂₈O₇ | 404,17623 |
| CBTT | C₂₁H₃₀O₅ | 362,20205 | CBTTA | C₂₂H₃₀O₇ | 406,19188 |
| Cannabichromanone B-C5 | C₂₁H₃₀O₅ | 362,20205 | Cannabichromanonic acid B-C5 | C₂₂H₃₀O₇ | 406,19188 |
| 8,9-Di-OH-CBT | C₂₁H₃₀O₅ | 362,20205 | 8,9-Di-OH-CBTA | C₂₂H₃₀O₇ | 406,19188 |
| (±)-4-acetoxycannabichromene-C5 | C₂₃H₃₂O₄ | 372,22278 | (±)-4-acetoxycannabichromenic acid-C5 | C₂₄H₃₂O₆ | 416,21261 |
| 2-acetoxy-6-geranyl-3-n-pentyl-1,4-benzoquinone-C5 | C₂₃H₃₂O₄ | 372,22278 | 2-acetoxy-6-geranyl-3-n-pentyl-1,4-benzoquinonic acid-C5 | C₂₄H₃₂O₆ | 416,21261 |
| 11-OAc-Δ⁹-THC | C₂₃H₃₂O₄ | 372,22278 | 11-OAc-Δ⁹-THCA | C₂₄H₃₂O₆ | 416,21261 |
| 5-acetyl-4-hydroxycannabigerol-C5 | C₂₃H₃₄O₄ | 374,23843 | 5-acetyl-4-hydroxycannabigerolic acid-C5 | C₂₄H₃₄O₆ | 418,22826 |
| 4-acetoxy-2-geranyl-5-hydroxy-3-n-pentylphenol-C5 | C₂₃H₃₄O₄ | 374,23843 | 4-acetoxy-2-geranyl-5-hydroxy-3-n-pentylphenolic acid-C5 | C₂₄H₃₄O₆ | 418,22826 |
| (-)-*trans*-CBT-Oet | C₂₃H₃₄O₄ | 374,23843 | (-)-*trans*-CBTA-Oet | C₂₄H₃₄O₆ | 418,22826 |
| SesquiCBG | C₂₆H₄₀O₂ | 384,29555 | SesquiCBGA | C₂₇H₄₀O₄ | 428,28538 |
| Carmagerol-C5 | C₂₃H₃₆O₆ | 408,24391 | Carmagerolic acid-C5 | C₂₄H₃₆O₈ | 452,23374 |
| 4-terpenyl cannabinolate-C5 | C₃₂H₄₂O₄ | 490,30103 |  |  |  |
| β-fenchyl-Δ⁹-tetrahydrocannabinolate C-5 | C₃₂H₄₆O₄ | 494,33233 |  |  |  |
| α-fenchyl-Δ⁹-tetrahydrocannabinolate C-5 | C₃₂H₄₆O₄ | 494,33233 |  |  |  |
| epi-bornyl-Δ⁹-tetrahydrocannabinolate C-5 | C₃₂H₄₆O₄ | 494,33233 |  |  |  |
| bornyl-Δ⁹-tetrahydrocannabinolate C-5 | C₃₂H₄₆O₄ | 494,33233 |  |  |  |
| α-terpenyl-Δ⁹-tetrahydrocannabinolate C-5 | C₃₂H₄₆O₄ | 494,33233 |  |  |  |
| 4-terpenyl-Δ⁹-tetrahydrocannabinolate C-5 | C₃₂H₄₆O₄ | 494,33233 |  |  |  |

| Table S2. List of possible oxidation products from CBD used for HRMS target analysis in cell lysates. | | |
| --- | --- | --- |
| Compound | Formula | Molecular weight |
| Cannabidiol (CBD) | C₂₁H₃₀O₂ | 314,21730 |
| CBD Monomeric Hydroxyquinone | C₂₁H₂₈O₃ | 328,2038 |
| CBD Dimeric Hydroxyquinone-A | C₄₂H₅₆O₆ | 656,4077 |
| CBD Dimeric Hydroxyquinone-B | C₄₃H₅₈O₆ | 670,4233 |

CBDA

CBGA

CBG

CBD

CBN

CBG

CBD

CBDA

CBGA

CBN

CBG

CBD

CBN

CBG

CBD

A

B

C

D

**Figure S1:** HPLC-UV chromatograms of R2 (A), D2 (B), R3 (C) and D3 (D) extracts recorded at 220 nm. The main neutral and acid cannabinoids quantified in the extracts are shown.


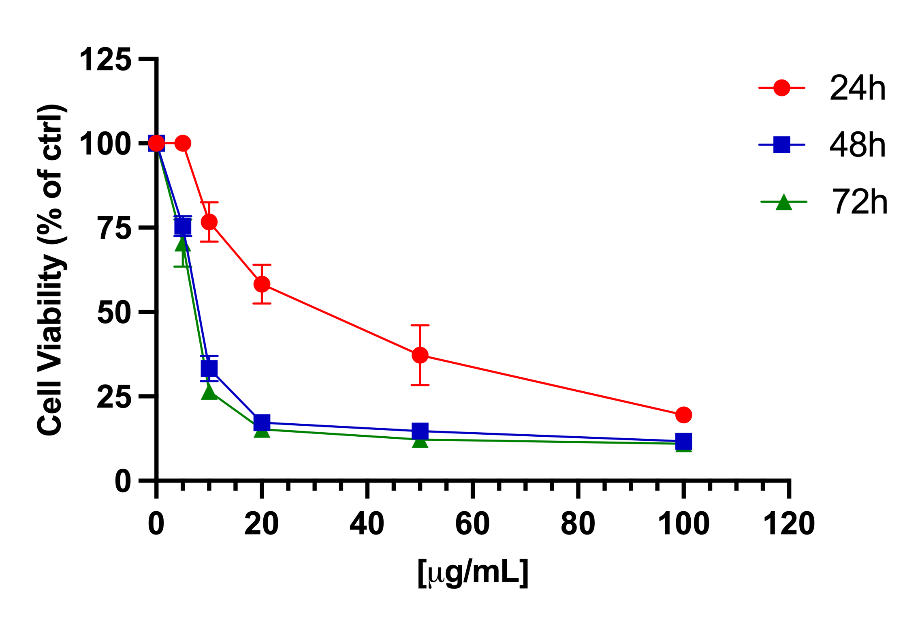


**Figure S2**: Dose/response curves of the D1 extract at 5-100 µg/mL at different time points (24, 48, 72 h). Data in the graph are shown as mean ± SD (n = 4 per group).


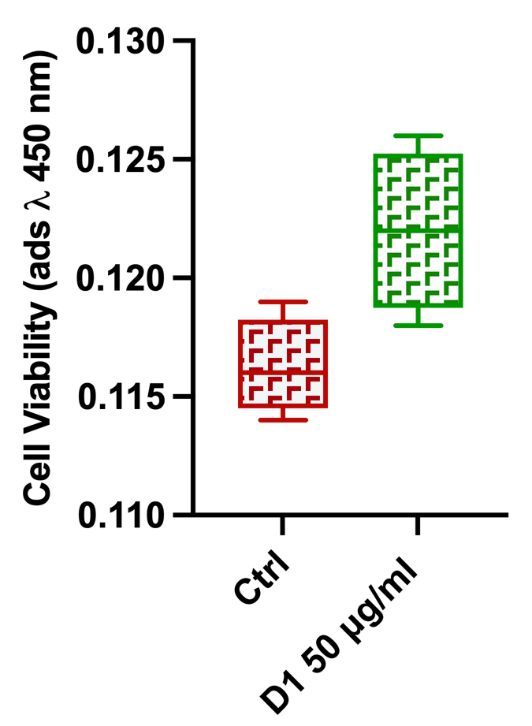


**Figure S3**: Effect of the D1 extract 50 µg/mL after 48 h of incubation on human peripheral blood mononuclear cells (PBMCs). Data in the graph are shown as mean ± SD (n = 4 per group).


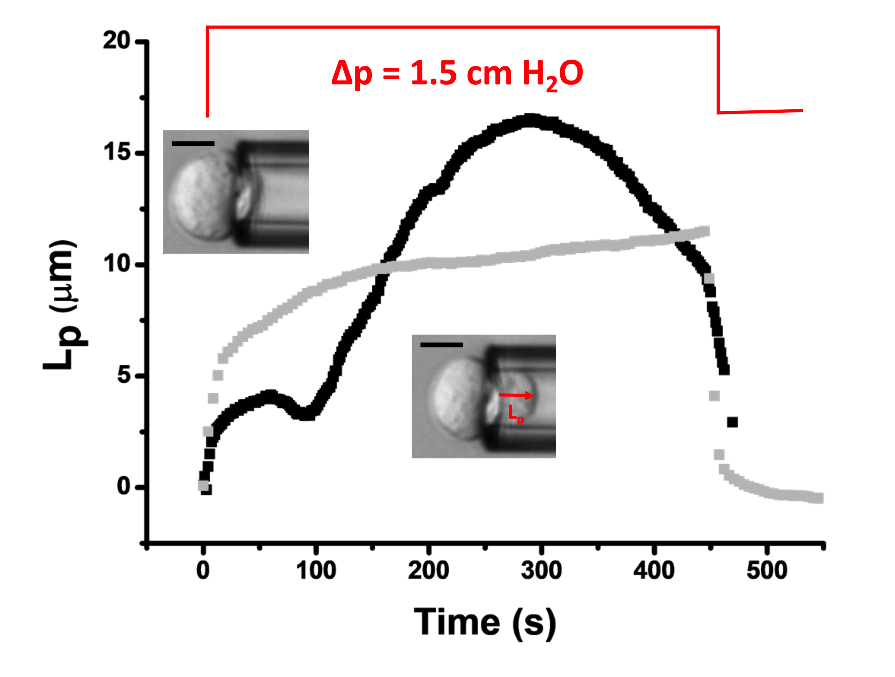


**Figure S4**: Representative curves for the aspiration (due to a pressure step of 1.5 cm H2O – red curve) of control K562 cells (gray line) and K562 cells exposed to D1 at 30 μg/mL. Lp represents the length of the cell tongue inside the micropipette. The two images (bar = 10 μm) show a K562 cell before the application of the pressure step and during the aspiration process.
